# Effects of Different Polysorbates (Tween) on Recombined Whipped Cream: Interfacial Properties, Stability, and Aeration Properties

**DOI:** 10.3390/foods15111878

**Published:** 2026-05-26

**Authors:** Yingjun Zuo, Guosen Yan, Yuru Wang, Yan Li

**Affiliations:** 1 Beijing Engineering and Technology Research Centre of Food Additives, School of Food and Health, Beijing Technology and Business University, Beijing 100048, China; wxsxhzhn@163.com; 2 Institute of Food Science and Technology, Chinese Academy of Agricultural Sciences, Beijing 100193, China; guosenyan1@163.com; 3 College of Food Science and Nutritional Engineering, China Agricultural University, Beijing 100083, China; wangyurucau@163.com

**Keywords:** polysorbate, interface behavior, whipping cream, aeration properties

## Abstract

Emulsifiers play a crucial role in improving the shelf-life stability and aeration quality of recombined whipped cream (RDC). This study systematically investigated the interfacial adsorption behaviors of four types of polysorbates (Tween20, Tween40, Tween60, Tween80) at concentrations ranging from 0.025% to 0.1%, as well as their impacts on the emulsion properties, interfacial composition, stability, and whipping properties of RDC. The results revealed that all four polysorbates significantly reduced the oil/water (O/W) interfacial tension and decreased the fat globule size, with these effects intensifying as the concentration increased. At equivalent concentrations, Tween20 exhibited the strongest particle size-reducing capacity and induced the most pronounced interfacial protein displacement, yet the compromised mechanical strength of the interfacial film led to diminished emulsion stability. In contrast, the RDC sample incorporated with Tween40 displayed the optimal emulsion stability. Moreover, all four polysorbates markedly shortened the whipping time and enhanced the overrun, but simultaneously increased serum loss. Notably, Tween80, which contains unsaturated fatty acid chains, contributed to the enhancement of foam firmness at high concentrations (0.075–0.1%), which may be correlated with its balanced properties of interfacial strength and interfacial tension. These findings facilitate a deeper mechanistic understanding of the relationship between the interfacial adsorption behavior of emulsifiers and the emulsion stability and aeration performance of RDC emulsions.

## 1. Introduction

Recombined whipped cream (RDC) is an oil/water (O/W) emulsion consisting of anhydrous milk fat, concentrated milk protein, emulsifier, stabilizer, and water [1]. It can be transformed into a three-phase system via whipping and remains stable for a certain period. However, since the primary ingredient, anhydrous milk fat, lacks natural fat globule membranes, the system is not stable enough during storage and transport, necessitating additional emulsifiers to address this. Furthermore, RDC destabilization is necessary during whipping and aeration to allow the partial aggregation of fat globules. This creates a foam structure in which the protein-stabilized emulsion and the fat globules coexist. Since this three-phase system can remain stable for a certain time, finding the right balance between stability and destabilization is crucial for producing high-quality RDC [2].

Proteins are amphoteric molecules that can adsorb at the O/W interface of an emulsion to form a viscoelastic interfacial film, the properties of which determine the stability and agitation performance of the RDC [3]. Higher protein hydrophobicity and concentrations at the interface generally reduce the interfacial tension, while increasing both the interfacial film elasticity and emulsion stability [4]. Furthermore, the interfacial properties can also regulate the occurrence and development of fat aggregation during agitation and aeration, consequently affecting the agitation performance of the emulsion [5]. Emulsifiers function as surfactants, modifying interfacial properties by interacting with proteins to balance stability and aeration in the emulsion [6].

Polysorbates (Tween) are nonionic amphiphilic emulsifiers that have a hydrophilic group of polyoxyethylene chains and a hydrophobic group of fatty acid hydrocarbon chains [7]. These emulsifiers exhibit low critical micelle concentrations (CMC), as well as good thermal and hydrolytic stability [8]. Polysorbate 20, 40, 60, and 80, which are approved for use in cream, share the same hydrophilic head structure but have different fatty acid chains. Polysorbate 80 contains a carbon–carbon double bond, presenting an unsaturated fatty acid chain, while the other three present saturated fatty acid chains. Synergistic and competitive adsorption represent the main modes of interfacial interaction between polysorbate and protein [9]. According to “mountain theory,” synergistic adsorption involves the formation of a mixed adsorption layer of protein and polysorbate at the interface, where polysorbate adsorbs on the weaker areas of the protein membrane. Competitive adsorption occurs when both polysorbate and protein are present, with polysorbate capable of replacing the protein in the adsorption layer. As the polysorbate concentration increases, it gradually replaces the protein at the interface, resulting in a looser, thinner adsorption layer [10,11,12]. Therefore, polysorbate effectively reduces interfacial tension, which promotes emulsion formation and stability [13].

Current research on polysorbates in whipped cream systems mostly focuses on optimizing emulsifier formulations. Minimal systematic studies are available on the structure, interfacial properties, and relationship between stability and emulsifying/foaming properties of polysorbates. Therefore, this work used anhydrous butter and milk protein concentrate 70 (MPC70) as raw materials to assess the impact of four distinct polysorbates (Tween20, Tween40, Tween60, and Tween80) on the stability and whipping properties of whipped cream with a fat content of 36%. This research provides a theoretical basis and product parameters for developing stable, high-quality RDC.

## 2. Materials and Methods

### 2.1. Materials

The AMF was obtained from Anchor (Auckland, New Zealand), while the MPC70 was supplied by Saishang Dairy Industry (Ningxia, China), and the Tween 20 (T20), Tween 40 (T40), Tween 60 (T60), and Tween 80 (T80) were provided by Longsha (China).

### 2.2. Preparation of RDC

Based on the critical micelle concentration [14] and regulatory considerations (<0.1%), Tween was added in a gradient within the 0–0.1% concentration range to prepare RDC. Different Tween mass fractions (0%, 0.025%, 0.05%, 0.075%, and 0.1%) were added to the MPC solution as the aqueous phase, while melted AMF was used as the oil phase. The two phases were mixed and diluted with water to a final sample containing 36% fat and 1% protein. The mixture was stirred at 700 rpm for 30 min at 70 °C. Next, the emulsion was blended at 6 MPa using a two-stage homogenizer and stored at 4 °C for 24 h.

### 2.3. Emulsion Properties

#### 2.3.1. Particle Size Distribution

A suitable quantity of the stored sample was diluted 10-fold, after which the particle size was determined via the wet method using an LS 230 laser particle size analyzer (Beckman Coulter, Brea, CA, USA). The mixture dropped slowly into the sample cell, maintaining an occlusion level between 8% and 12%. The instrument parameters included a stirring speed of 50%, a particle density of 1 g/mL, and an absorbance of 1.46, while the dispersed phase consisted of deionized water with a refractive index of 1.333. Each sample was measured in triplicate, and the value was averaged. D_[3,2]_ represents the particle size of the sample.

#### 2.3.2. Shear Viscosity

The shear viscosity of the sample was determined using a viscometer (DV-III, Brookfield, WI, USA). Here, 8 g of the sample stored at 4 °C for 24 h was placed in a container and measured using an SC4-18 rotor at a rotation speed of 30 rpm/min, a test time of 4 min, and a measurement point interval of 4 s.

#### 2.3.3. ζ-Potential

The stored sample was diluted 500-fold with deionized water and slowly added to a dedicated sample cell (DTS1070) to avoid bubble formation. A Malvern ZetaSizer laser particle size analyzer (Malvern Panalytical, Malvern, UK) was used to measure the ζ-potential at a temperature of 25 °C and an equilibration time of 3 min. Each sample was measured three times.

#### 2.3.4. Physical Stability

The emulsion stability was determined using a LUMiSizer dispersion stability analyzer (LUM GmbH, Berlin, Germany). A 0.5 mL sample was transferred into a LUMI tube (model: PC-110-131XX) to the graduation mark and balanced. The instrument was preheated to 25 °C, and the sample was placed in the slot of the centrifuge tray. Next, 241 measurements were performed at a test temperature of 25 °C, a centrifugation speed of 4000 rpm/min, a light factor of 1.00, and a measurement interval of 30 s.

### 2.4. Thermodynamic Behavior

The crystallization and melting behavior of the milk fat in the RDC were analyzed using a differential scanning calorimeter (TA Q200 DSC, TA Instruments, New Castle, DE, USA). The crystallization temperature, crystallization peak temperature, enthalpy and other thermal properties of samples were determined by analyzing their non-isothermal crystallization and melting processes. Accurately weigh 5 mg of sample into a standard aluminum pan matched with the instrument, and equilibrate at an initial temperature of 40 °C for 2 min. Subsequently, the temperature was decreased to −10 °C at a cooling rate of 5 °C/min to investigate the exothermic crystallization process. Afterwards, the sample was heated up to 45 °C at a heating rate of 5 °C/min to characterize the endothermic melting behavior. 

### 2.5. Interfacial Protein Concentration

The protein concentrations on the surfaces of the fat globules in the sample were determined using a method delineated by Yan [15], with modifications. A 10 g sample was placed in a 50 mL tube, centrifuged at 10,000 rpm for 1 h at 4 °C, and stored in a refrigerator at 4 °C. The fat layer was allowed to solidify, after which the supernatant and precipitate were removed to determine the protein content. The interfacial protein concentration was calculated using Formula (1).

(1) $$interfacial\;protein\;concentration=\frac{P_{0}-P_{s}}{M_{c}\times SSA}\times 10^{4}$$

where *P*_0_ is the total protein content, g; *P_S_* is the liquid phase protein content, g; *M_C_* is the emulsion layer mass, g; and *SSA* is the specific surface area of fat globules, cm^2^/g, obtained using a laser particle size analyzer.

### 2.6. Interfacial Adsorption Behavior

The adsorption behavior and interfacial tension of the proteins at the O/W interface were determined using an interfacial tensiometer (Theta Flex, Biolin Scientific AB, Västra Frölunda, Sweden). A 1% protein solution mixed with polysorbate was used as the aqueous phase, while AMF purified using Florisil was used as the oil phase. The aqueous phase was placed in a syringe, after which approximately 4.5 g of oil phase was added to the sample dish. The aqueous phase was immersed in the oil phase and tested using the hanging drop method. The interfacial tension was calculated based on the Yang-Laplace equation, utilizing One Attension software (Version 4.0). The test was performed at 42 °C, with interfacial tension stabilization denoting the endpoint. Before the measurements, the AMF was purified using Florisil to prevent the impurities in the anhydrous oil from affecting the interfacial tension.

### 2.7. Oscillatory Dilatational Rheology

The oscillating drop module of a Theta Flex interfacial tensiometer was used to determine the interfacial viscoelastic modulus. The parameters included a frequency of 0.1 Hz, an amplitude of 0.7 Hz, and a recording time of eight 120 s cycles.

### 2.8. Whipping Properties

#### 2.8.1. Whipping Time

The sample was refrigerated at 4 °C for 3 d. Then, approximately 200 g of heavy cream was placed in an iron bowl and whipped in a 4 °C ice-water bath at low speed for the first 30 s, and then at the highest speed until reaching the endpoint. The time required to reach the endpoint was recorded.

#### 2.8.2. Overrun

The overrun was calculated by measuring the amount of whipped cream at the end of whipping using Formula (2).

(2) $$Overrun\left(\%\right)=\frac{m_{1}-m_{2}}{m_{2}}\times 100\%$$

where *m*_1_ is the mass of the same volume of unwhipped cream, g and *m*_2_ is the mass of the same volume of whipped cream, in g.

#### 2.8.3. Serum Loss

Approximately 5 g of the whipped cream was passed through a 30-mesh sieve (5 cm in diameter), while the mass of the cream (*m*_1_) was recorded. The sieve was placed in a plastic cup and incubated at 25 °C for 2 h. The liquid leaking from the plastic cup (*m*_2_) was recorded, and the serum loss was calculated using Formula (3).

(3) $$Serumloss\left(\%\right)=\frac{m_{2}}{m_{1}}\times 100\%$$

#### 2.8.4. Firmness

A whipped cream sample was placed in a piping bag, which was used to fill a flat-bottomed beaker, ensuring no gaps remained. Then, a TA texture analyzer (HDP/FE3) was used to determine the firmness at a probe speed of 10 mm/min, a measurement distance of 40 mm, and a trigger force of 0.5 N.

### 2.9. Confocal Laser Scanning Microscopy

Here, 2 mL each of 0.02% (*w*/*t*) NR and FITC staining solutions were added to a 200 g sample, followed by stirring and aeration. A slide was immediately prepared for observation using an argon lamp as the excitation light source and an excitation wavelength of 488 nm. The receiving wavelength is detected using a photomultiplier tube, with a range of 500 nm to 536 nm for FITC and 595 nm to 648 nm for NR.

### 2.10. Statistical Analysis

The data were expressed as the mean ± standard deviation. Data analysis was performed using IBM SPSS Statistics 26.0. One-way ANOVA employed the Duncan (homogeneous variance) and Dunnett’s T3 (non-homogeneous variance) methods to compare statistical significance. Laser confocal microscopy images were processed and analyzed using ImageJ software (version 1.53t). A *p*-value < 0.05 was considered statistically significant.

## 3. Results and Discussion

### 3.1. The Effect of Polysorbate on the Physical Properties of the RDC Emulsions

#### 3.1.1. Particle Size Distribution

**Figure 1:**
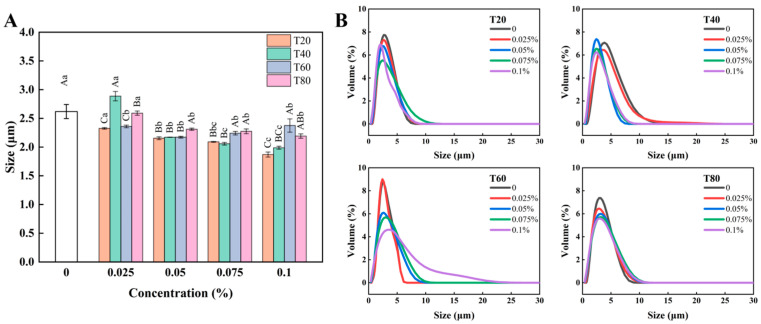
The effect of the polysorbate type and concentration on the (**A**) average particle size and (**B**) particle size distribution in the RDC emulsions. Lowercase letters indicate differences between the concentrations of the same polysorbate, while uppercase letters denote variations between the different polysorbates at the same concentration.

The fats in RDC emulsions are dispersed in the aqueous phase as fat globules. The adsorption of emulsifiers and proteins to the globule surfaces leads to film formation that reduces the interfacial tension and creates steric hindrance and physical barriers [16], consequently maintaining emulsion stability [17]. Figure 1A shows the impact of the different types and addition amount of polysorbates on the average particle size of the fat globules. All four polysorbates reduced the fat globule size. The average particle size decreased as the added concentration increased until reaching the CMC, after which it stabilized. The particle size distribution (Figure 1B) revealed a unimodal pattern across all samples, confirming uniform droplet sizes. At low concentrations (0.025%), the average particle sizes of T20, T40, and T80 did not differ significantly from those of the control group. As the added quantity increased (0.05–0.1%), the average particle sizes of the samples declined gradually (*p* < 0.05). This was likely due to the weak interfacial adsorption of the emulsifier at low concentrations, where proteins still dominated the interface. Higher polysorbate concentrations were more successful at reducing the interfacial tension, which helped decrease the droplet size [18]. Notably, T60 significantly reduced the fat globule size at a concentration of 0.025% (*p* < 0.05), indicating a lower CMC. As the T60 concentration rose to 0.075%, the droplet size began to increase, also elevating the half-maximum width of the particle size distribution. This suggests the presence of partial aggregation or agglomeration that contributes to the increase in droplet size.

The different polysorbates exhibited varying efficacy in reducing the size of the fat globules at the same concentration. A comprehensive analysis of the minimum concentrations of the four polysorbates showed that their ability to minimize fat globule size ranked as follows: T20 > T40 > T60 > T80. Such discrepancies are attributed to the synergistic action of molecular structure, HLB value and interfacial adsorption kinetics. T20 had the shortest fatty acid chain (lauric acid with 12 carbons) [19], which yielded the largest hydrophilic-lipophilic balance (HLB) value of 16.7. Therefore, T20 displayed the highest hydrophilicity, enhancing its solubility and migration efficiency in the aqueous phase, which enables it to rapidly form stable small-sized droplets during shearing. Similarly, Ananingsih [20] systematically compared the ability of T20, T60, and T80 to stabilize nanoemulsions. The results indicated that T20 produced smaller, more uniform droplets, which was attributed to its superior hydrophilicity and interfacial adsorption kinetics.

#### 3.1.2. Shear Viscosity

**Figure 2:**
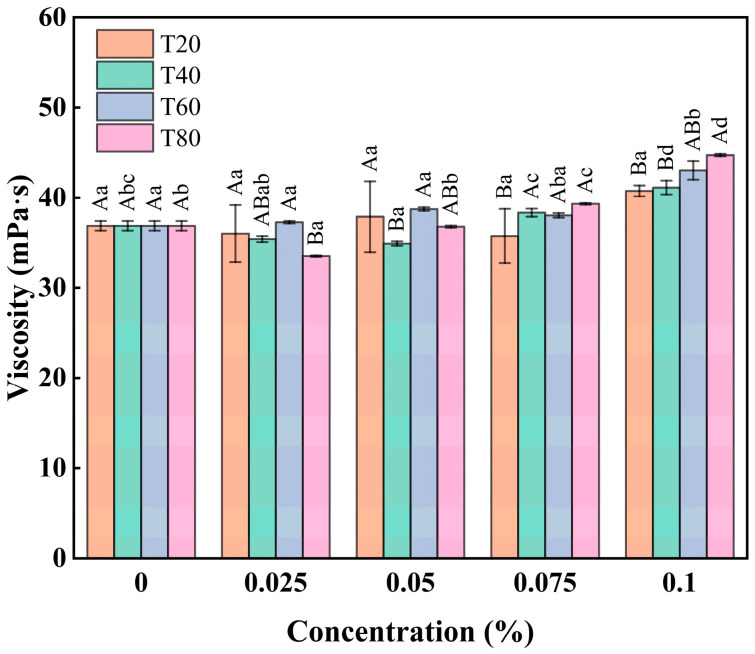
The effect of the polysorbate type and concentration on the shear viscosity of the RDC emulsions. Lowercase letters indicate differences between the concentrations of the same polysorbate, while uppercase letters denote variations between the different polysorbates at the same concentration.

Shear viscosity is an important indicator for evaluating the properties of emulsions. Stokes’ law states that higher viscosity increases the force that fat globules must overcome to move, while decreasing particle speed and enhancing stability [21]. As shown in Figure 2, the viscosity of the cream increased at T40 and T60 concentrations of 0.1% while the T80 concentration peaked at 0.075% (*p* < 0.05). This was consistent with the particle size results. Smaller particles reduced the distance between the fat globules, while stronger interactions amplified the energy required for their movement, which increased the viscosity [22]. A study by Fuller et al. [23] showed that polysorbates increased the shear sensitivity of fat globules, leading to partial aggregation and higher shear viscosity. The medium-chain polysorbates (T20) did not affect the shear viscosity of the recycled cream (*p* > 0.05), while the long-chain polysorbates T40, T60, and T80) increased the shear viscosity of the system. This may be because the fatty acid chains of T20 are relatively short, reducing the interfacial reinforcement effect (see Section 3.2 for details). Therefore, the changes in the aggregation state of milk fat globules or the microstructure of the continuous phase were insufficient to statistically modify the shear viscosity.

#### 3.1.3. ζ-Potential

**Figure 3:**
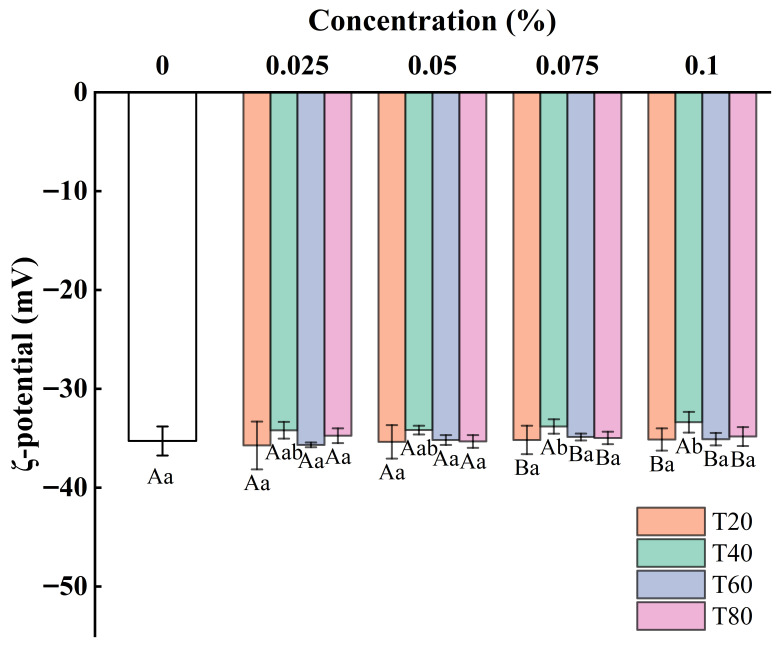
The effect of the polysorbate type and concentration on the zeta potential of the RDC emulsions. Lowercase letters indicate differences between the concentrations of the same polysorbate, while uppercase letters denote variations between the different polysorbates at the same concentration.

Droplets acquire their charge via the adsorption of charged or easily ionized substances on their surfaces, such as proteins, polysaccharides, and ionic surfactants. The ζ-potential represents the potential of the shear plane, reflecting the charge intensity that influences the attraction or repulsion between particles. As shown in Figure 3, no statistical differences were evident between the ζ-potential values of T20, T60, and T80 and the blank sample (*p* > 0.05). The absolute ζ-potential decreased at an added T40 concentration of ≥0.075% (*p* < 0.05), after which it stabilized (*p* > 0.05). Although polysorbate is an uncharged nonionic emulsifier, replacing interfacial proteins may modify the charge properties. According to the “mountain-building” principle, when emulsifiers compete with interfacial proteins for adsorption, they tend to first adsorb into the spaces between the protein molecules [18]. Therefore, it is speculated that T40 mainly adsorbs in the interprotein spaces at low concentrations and does not change the ζ-potential. As the concentration increased (≥0.075%), more T40 was adsorbed on the interface, leading to the detachment and disintegration of the interfacial protein layer. This reduced the interfacial protein concentration (Figure 6), the electrostatic interaction, and charge strength.

#### 3.1.4. Physical Stability

**Figure 4:**
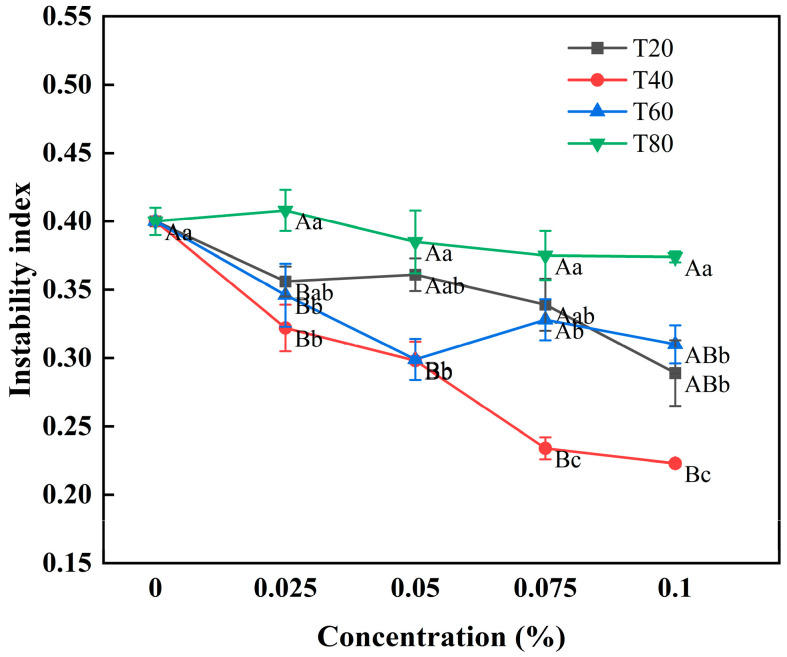
The effect of the polysorbate type and concentration on the instability index of the RDC emulsions. Lowercase letters indicate differences between the concentrations of the same polysorbate, while uppercase letters denote variations between the different polysorbates at the same concentration.

Centrifugation was used to simulate particle migration during the long-term storage of emulsions, while near-infrared spectroscopy was employed to record the sedimentation process. Finally, the stability of the system was quantified using the instability index, with a smaller value indicating greater stability [24]. Figure 4 shows the effect of different polysorbates and their concentrations on sample stability. The instability index generally declined as the polysorbate concentration increased, indicating that polysorbate addition enhanced system stability. Compared with the blank sample, both the 0.1% T20 and 0.025% T40 concentrations significantly reduced the system instability index (*p* < 0.05). Although no statistically significant differences were observed between the values obtained at 0.025% and 0.05% concentrations (*p* > 0.05), a concentration of 0.075% further reduced the instability index (*p* < 0.05). The instability index decreased at a T60 concentration of 0.025% (*p* < 0.05), while no further decline was evident beyond this point (*p* > 0.05). The instability index of the T80 emulsified system decreased slightly, but the difference was not statistically significant (*p* > 0.05). In summary, the ability to improve emulsion stability ranked as follows: T40 > T60 > T20 > T80.

### 3.2. The Effect of Polysorbate on the Thermodynamic Properties of the RDC Emulsions

**Figure 5:**
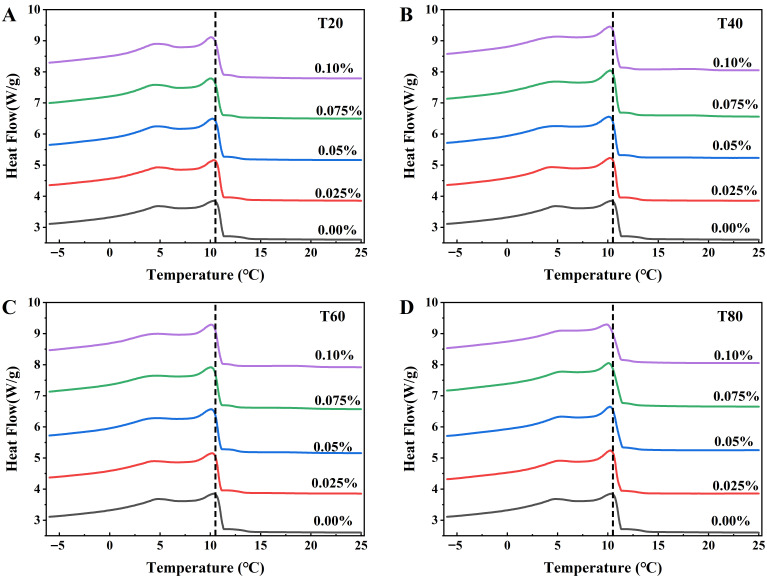
The effect of the polysorbate type and concentration on the thermodynamic properties of the RDC emulsions. (**A**) T20. (**B**) T40. (**C**) T60. (**D**) T80.

The effect of varying added polysorbate concentrations on the crystallization behavior of milk fat was determined using differential scanning calorimetry (DSC). As shown in Figure 5, all samples exhibited two sharp exothermic crystallization peaks, showing uniform shapes and widths, with no multiple peaks or broadening. The primary crystallization peaks of all the samples were concentrated in a range of 9–11 °C (Figure S1). This indicated that the addition of polysorbate did not significantly alter the crystallization behavior of the milk fat in the concentration range of this study (0.025–0.1%), with crystallization polymorphism predominantly governed by the β′-crystal form. This may be because polysorbate is mainly distributed in the continuous phase and oil–water interface, rather than inside the droplets, preventing it from effectively participating in fat crystal nucleation and growth [25]. Therefore, it has no significant regulatory effect on the overall crystallization behavior, polymorphism transformation, and solid fat content of milk fat. Notably, the peak temperatures of all the samples declined as the polysorbate concentration increased, while the endothermic peaks decreased in the 11–14 °C range. At a 0.05% concentration, T40 and T60 significantly reduced the crystallization peak temperature to 10.07 °C and 10.12 °C, respectively, while a 0.075% T20 concentration substantially decreased the crystallization peak temperature to 10.125 °C (*p* < 0.05). This may be because the polysorbate fatty acid chains disrupt the fat molecule arrangement. This hinders crystal formation, requiring higher supercooling for crystallization [26]. Han et al. reported an analogous phenomenon upon the supplementation of hydrophilic polyglycerol fatty acid ester (HLB 16) into palm stearin [27].

### 3.3. The Effect of Polysorbate on the Interfacial Behavior of the RDC Emulsions

#### 3.3.1. Interfacial Protein Concentration

**Figure 6:**
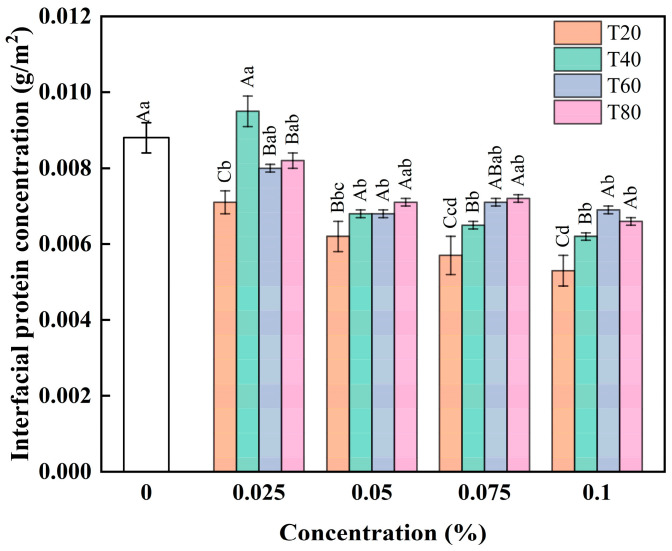
The effect of the polysorbate type and concentration on the interfacial protein concentration in the RDC emulsions. Lowercase letters indicate differences between the concentrations of the same polysorbate, while uppercase letters denote variations between the different polysorbates at the same concentration.

The interfacial protein layer that forms after protein adsorption at the O/W interface is crucial for maintaining fat globule stability. However, the added emulsifier competes with the interfacial protein for adsorption, changing its concentration and properties [28]. As shown in Figure 6, all four polysorbates significantly reduced the interfacial protein concentration. T20 concentrations between 0% and 0.1%, as well as T40, T60, and T80 concentrations of 0.05%, decreased the interfacial protein level (*p* < 0.05). However, as these concentrations continued to rise, no significant changes were evident in the interfacial protein content. This indicated that the interfacial concentration limited the ability of the polysorbates to replace the interfacial proteins. The fatty acid-carbon chains of T20, T40, T60, and T80 gradually increased. The hydrophobic chain of T80 contained an unsaturated double bond, which gradually weakened its hydrophilicity. Studies have shown that the stronger the hydrophilicity of the emulsifier, the stronger its ability to replace interfacial proteins [29,30]. This behavior is consistent with the principles of orogenic displacement theory. At low concentrations, polysorbate molecules initially occupy the gaps between interfacial protein molecules. Here, no significant changes were evident in the interfacial protein concentration, and the interfacial layer mainly consisted of a protein network, while the adsorbed emulsifier helped reduce interfacial tension. As the concentration increased, polysorbate molecules directly competed with proteins for adsorption sites, which decreased the protein adsorption area. Furthermore, the polysorbate molecules in the spaces between the proteins gradually exerted pressure on them, causing interfacial protein bulging and deformation. A further increase in the concentration ultimately led to the desorption of the bulging proteins from the interfacial layer, significantly decreasing the interfacial protein concentration [28]. Due to its high hydrophilicity and smaller molecular size, T20 more effectively penetrated the gaps in the protein network, demonstrating the strongest protein-displacement ability.

The concentration of interfacial proteins showed a significant positive correlation with particle size. This relationship is not simply due to a single competitive adsorption effect. Instead, it is influenced by multiple factors, including lower interfacial tension, variations in adsorption kinetics, and changes in the interfacial film properties. During homogeneous shearing, polysorbate diffuses much faster than milk proteins since the diffusion rate of small molecules is inversely proportional to the square root of the molecular weight. This allows it to preferentially adsorb at the oil–water interface, which rapidly reduces interfacial tension and allows shear forces to break large droplets into smaller ones. During droplet formation, proteins near the interface quickly adsorb onto the interfacial layer due to diffusion time and migration rate constraints. The interfacial adsorption of polysorbates is a dynamic process. When the polysorbate concentration is sufficiently high, it gradually replaces proteins on the interface, forming a polysorbate-dominated interfacial film. This hybrid film exhibits much lower interfacial tension than a pure protein film, enabling the formation of smaller droplets with lower energy input and more effectively preventing droplet re-aggregation after breakup. Therefore, our results indicate that a lower interfacial protein concentration correlates with a higher polysorbate proportion at the interface. This results in a more significant reduction in interfacial tension and, ultimately, smaller fat globule sizes. In the same homogeneous conditions (i.e., the same adsorption time), polysorbates with smaller molecular weight and faster adsorption rate occupy the adsorption sites of proteins, which significantly decreased the protein concentration at the interface. Additionally, polysorbates more efficiently reduced interfacial tension, which substantially reduced the fat globule size. Therefore, our results showed that a lower interfacial protein concentration correlated with smaller particle sizes. However, polysorbate forms a thinner, less robust interfacial layer compared to protein interactions, resulting in a significantly weaker mechanical barrier. This explains why T20 effectively reduces the fat globule size, but does not significantly improve the viscosity and instability index [29].

#### 3.3.2. Interfacial Tension

**Figure 7:**
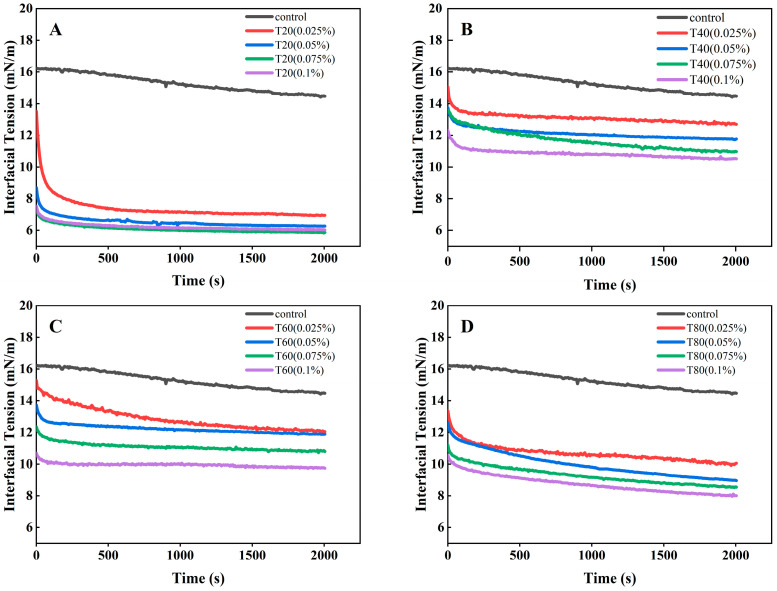
The effect of the polysorbate type and concentration on the interfacial tension of the RDC emulsions. (**A**) T20. (**B**) T40. (**C**) T60. (**D**) T80.

Interfacial tension reflects how polysorbates interact at the O/W interface and impact interfacial tension. Figure 7 shows the changes in the interfacial tension in the emulsions after treatment with different polysorbates. All four polysorbates significantly reduced interfacial tension as their concentrations decreased. Injecting the aqueous phase into the oil phase caused rapid protein and polysorbate molecule diffusion and adsorption at the O/W interface. The adsorption behavior of small molecules at oil–water interfaces consists of diffusion, penetration/spreading and molecular rearrangement [31]. Given that rapid diffusion and interfacial adsorption occur instantly upon droplet generation, the diffusion stage is hardly detectable. The initial interfacial tension can serve as an indirect indicator of diffusion rate. During the initial stage, the interfacial tension of the control group without polysorbate was 16.22 mN/m, which was significantly reduced by the addition of polysorbate. The interfacial tension of T20, T40, and T80 was all below 13.59 mN/m. This may be because the polysorbate displays a lower molecular weight than the protein, accelerating the diffusion rate and enhancing the surface area occupancy at the same concentration [18]. Among them, the sample supplemented with T20 presented the lowest initial interfacial tension, which could be attributed to its lowest molecular weight and highest HLB value accompanied by superior hydrophilicity. Over time, the interfacial tension stabilized in the later stage, indicating that molecular adsorption at the interface reached equilibrium. At higher concentrations, polysorbate gradually replaced protein at the interface, enhancing activity and reducing interfacial tension and the time required to reach adsorption equilibrium. The lower interfacial tension reduced the intermolecular forces and interfacial free energy, which promoted the formation of smaller droplets (Figure 1A). This was consistent with the particle size measurement results [32].

Different types of polysorbates vary in their ability to reduce interfacial tension. T20 was most effective at reducing the interfacial tension, showing a significant decrease to 6.98 mN/m, compared with the control group value of 16.22 mN/m. In a range of 0–0.1%, the interfacial tension values of the different samples ranked as follows: T20 < T80 < T40 ≈ T60. This is likely related to the length of the carbon chain at the tail [33]. T20 may have a higher CMC, enabling it to form a denser interface at the same concentration, thereby enhancing its ability to decrease surface tension. In addition, T80 may display stronger hydrophobicity than T60 due to the carbon–carbon double bonds in the fatty acid chain. This structural difference allows T80 to outperform T60 in reducing interfacial tension.

#### 3.3.3. Oscillatory Dilatational Rheology

**Figure 8:**
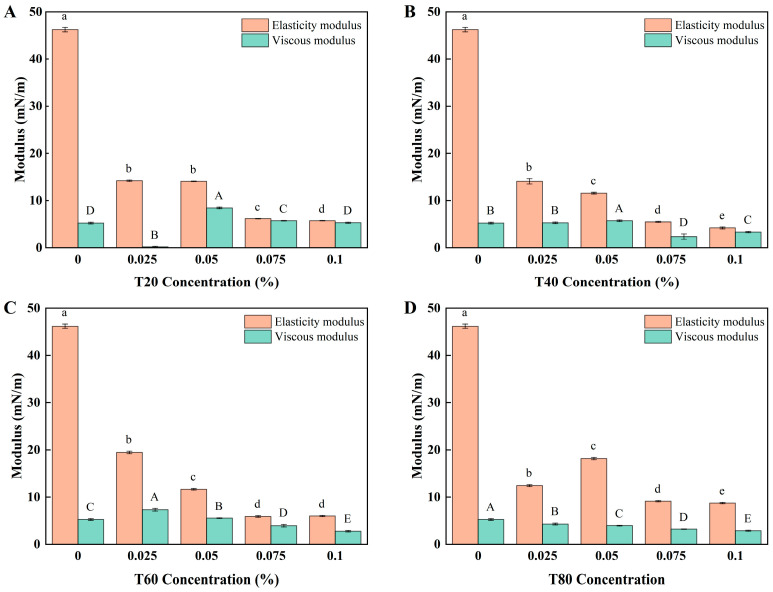
The effect of the polysorbate type and concentration on the interfacial modulus of the RDC emulsions. (**A**) T20. (**B**) T40. (**C**) T60. (**D**) T80. Different letters indicate statistically significant differences between samples (*p* < 0.05).

The elastic and viscous moduli of the interfacial layer were obtained by applying regular expansion oscillations to the droplets and detecting changes in the interfacial rheological properties, reflecting the mechanical properties of the interfacial layer [12,34]. Figure 8 shows the changes in the interfacial modulus of the solution based on the type and amount of the added polysorbate. All samples showed E′ > E″, indicating that the interfacial layer exhibited a mechanical structure with notable elasticity. The interfacial film of the blank group consisted entirely of a protein network and exhibited the highest elastic modulus of 46.2 mN/M. This was related to factors such as the electrostatic and hydrophobic interactions between proteins. These interactions increase resistance to the deformation of the interfacial layer and promote its restoration to its original structure. The addition of the four polysorbates significantly decreased the E′ of the interface layer, with further reductions observed as the concentration increased. This was because the simultaneous polysorbate and protein adsorption at the O/W interface weakened the protein interactions, which was consistent with findings by Zhang [12].

The interfacial elastic modulus is an essential parameter for determining the shear sensitivity and partial aggregation behavior of fat globules. Highly elastic pure-protein membranes resist shear deformation, which hinders fat crystal penetration and prevents partial fat globule aggregation. Conversely, interfacial membranes with low elasticity are fragile and prone to rupture under shear, leading to the excessive accumulation of fat globules. The findings indicated that polysorbate regulated the interfacial elastic modulus at different levels by controlling the interfacial protein concentration, which directly impacted the partial aggregation rate of the fat globules. At the same concentration, T20 exhibited a lower elastic modulus, which may be ascribed to its superior protein displacement capacity, consequently disrupting the continuity of the protein network within the interfacial film and forming more polysorbate-enriched domains. These regions displayed weaker intermolecular interactions and could not effectively resist interfacial deformation, which reduced the mechanical strength of the interfacial layer. However, no significant interfacial rheological changes (*p* > 0.05) were evident when the concentration was increased further (>0.075%). This may be because its ability to replace interfacial proteins and reduce interfacial strength is kinetically limited. Once a significant portion of the interfacial proteins had been replaced, further increasing the polysorbate concentration did not substantially impact the mechanical properties of the interfacial film. This directly influenced the rate of foam formation during subsequent stirring.

### 3.4. The Effect of Polysorbate on the Aeration Properties and Foam Microstructure of the RDC Emulsions

#### 3.4.1. Whipping Time

**Figure 9:**
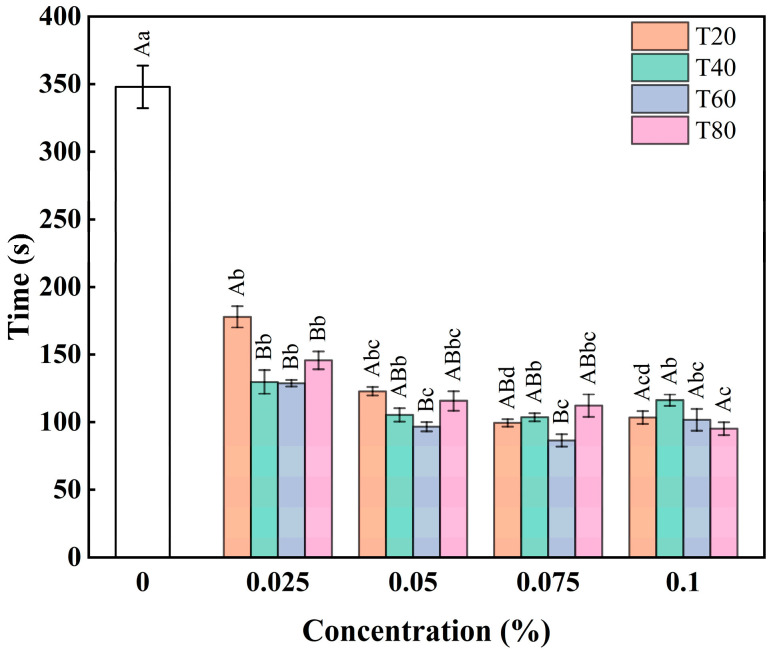
The effect of the polysorbate type and concentration on the whipping time of the RDC emulsions. Lowercase letters indicate differences between the concentrations of the same polysorbate, while uppercase letters denote variations between the different polysorbates at the same concentration.

The stirring properties of RDC emulsions are key to evaluating their application quality. The aeration process mainly includes three stages: rapid aeration, bubble encapsulation, and bubble stabilization [29,35]. The aeration time is mainly related to the second stage, depending on the rate at which emulsion droplets partially coalesce under mechanical action and adhere and diffuse at the air-water interface. Figure 9 shows the effect of adding different types and concentrations of polysorbates on the whipping time of the emulsion. Compared with the control group, the addition of all four polysorbates significantly reduced the whipping time of the emulsion, which decreased gradually as the polysorbate concentration increased. This phenomenon showed a significant positive correlation with changes in the interfacial elastic modulus (*p* < 0.05). A lower interfacial elastic modulus increased the shear sensitivity of the fat globules, promoting partial aggregation and continuous fat network formation on the bubble surface. The control group displayed a high interfacial elastic modulus of 46.2 mN/m, which hampered fat globule deformation and aggregation, resulting in the longest whipping time (348 s). The addition of 0.025% polysorbate significantly reduced the interfacial modulus (<20 mN/m), which dramatically decreased the whipping time. However, no changes were evident in the whipping time at polysorbate concentrations >0.05%, which was consistent with the analytical results of interfacial rheology. These results reveal that reducing the elastic modulus of the interfacial layer from 46.2 mN/m to 20 mN/m can markedly shorten the whipping time. When the elastic modulus is further decreased to below 10 mN/m, it exerts no significant influence on whipping time and may even deteriorate the stability of emulsions.

#### 3.4.2. Overrun

**Figure 10:**
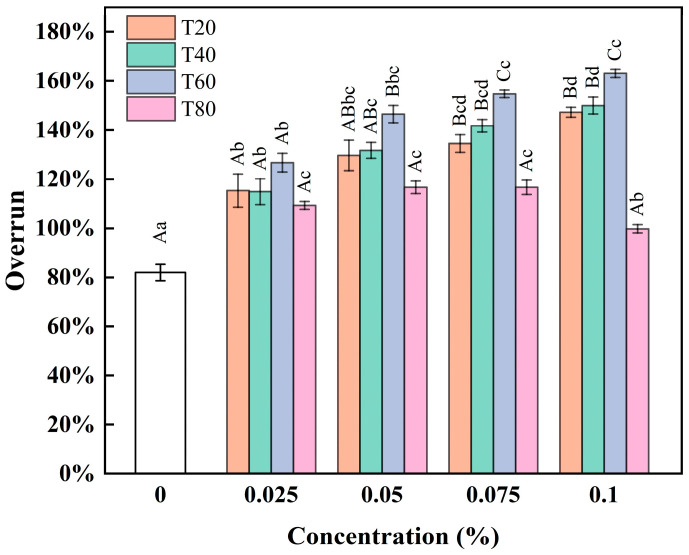
The effect of the polysorbate type and concentration on the overrun of the RDC emulsions. Lowercase letters indicate differences between the concentrations of the same polysorbate, while uppercase letters denote variations between the different polysorbates at the same concentration.

The overrun is the ability of the emulsion to encapsulate bubbles after aeration. As shown in Figure 10, the different types of polysorbates significantly improved the overrun. Added T20, T40, and T60 at 0–0.075%, 0–0.1%, and 0–0.05%, respectively, dramatically increased the overrun (*p* < 0.05), while that of T80 was relatively low at the same concentration. During the first stage of whipping, air was drawn into the cream, and the protein adsorbed onto the bubble surfaces [36]. Van Aken [37] proposed that the initial phase determined the foaming ability of light cream. During this stage, the control sample mainly utilized the protein in the liquid phase to capture and stabilize the bubbles. The addition of polysorbate acted with the protein to enhance bubble formation, which improved the overrun [38]. T80 displayed lower hydrophilicity compared to the other polysorbates, reducing its ability to stabilize bubbles and limiting an increase in the bubble count. Furthermore, existing studies have demonstrated that T80 tends to induce enhanced interfacial crystallization, which facilitates the partial coalescence of fat globules while impairing the air bubble entrapment capacity during whipping [39]. Overall, T60 exhibited the strongest ability to improve the overrun, followed by T20 and T40, with T80 being the least effective.

#### 3.4.3. Serum Loss

**Figure 11:**
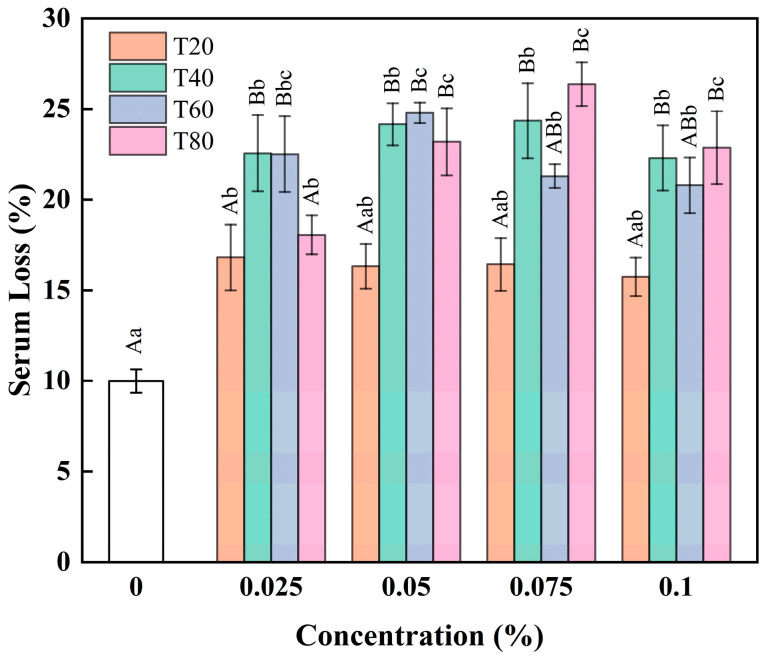
The effect of the polysorbate type and concentration on the serum loss of the RDC emulsions. Lowercase letters indicate differences between the concentrations of the same polysorbate, while uppercase letters denote variations between the different polysorbates at the same concentration.

Figure 11 shows the effect of the four types of polysorbates and their concentrations on foam stability. Overall, the polysorbates did not improve the serum loss of the emulsion, showing a higher value than the blank group. The results showed that all four polysorbates at a low concentration (0.025%) induced a significant increase in foam serum loss (*p* < 0.05). This could be explained by polysorbates increasing the number of air bubbles formed during whipping, which elevates the internal bubble content of the whipped cream, reduces its density at equivalent volume, and consequently weakens the network structure, rendering it unable to counteract gravity-induced serum loss. As polysorbate concentration elevated, the overrun showed a further significant increase, whereas serum loss exhibited no additional significant rise. This may be attributed to the synergistic regulation of serum loss by multiple factors including overrun, gas–liquid interface properties and network skeleton structure. Elevated polysorbate concentrations induce a marked decrease in interfacial protein concentration, which in turn reduces the mechanical strength of the interfacial layer. On the one hand, this may facilitate partial coalescence between fat globules and strengthen the supporting function of the network skeleton. On the other hand, proteins desorbed from the interface elevate the protein concentration in the continuous aqueous phase, allowing the formation of a robust protein interfacial layer at the gas–liquid interface during whipping, which acts as a mechanical barrier to stabilize air bubbles. Overall, in polysorbate-supplemented samples, there is a linear correlation between the reduction in interfacial protein concentration induced by competitive adsorption and serum loss. Specifically, the T20 sample, which had the lowest interfacial protein concentration, exhibited the lowest serum loss among the four polysorbate samples.

#### 3.4.4. Firmness

**Figure 12:**
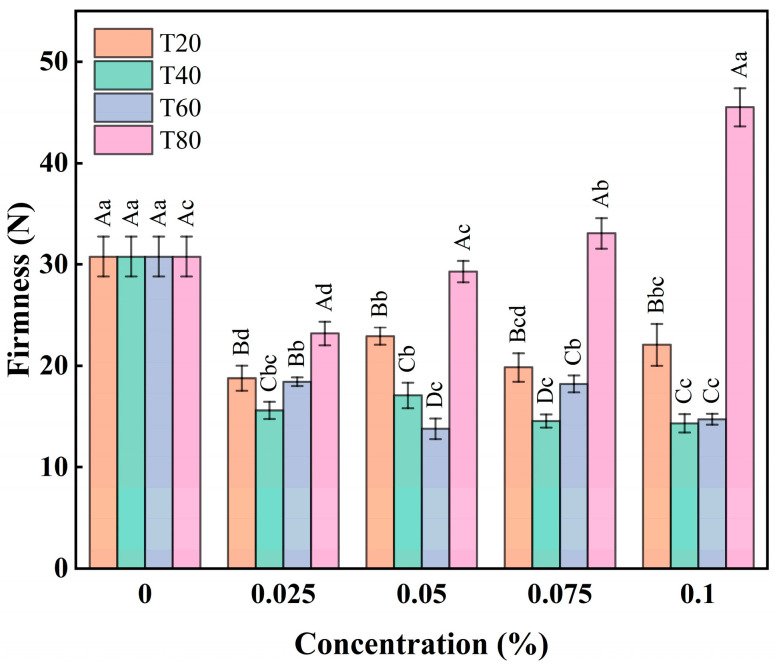
The effect of the polysorbate type and concentration on the firmness of the RDC emulsions. Lowercase letters indicate differences between the concentrations of the same polysorbate, while uppercase letters denote variations between the different polysorbates at the same concentration.

Inflation is an important indicator of the quality of the product, affecting the moldability and plasticity of the product. Figure 12 shows the effect of the different types of polysorbates and their concentrations on the firmness of the RDC emulsion foam. At low concentrations, T20, T40, and T60 significantly reduced the firmness (*p* < 0.05), while that of T80 demonstrated an initial decline at concentrations of 0.25–0.05%, followed by a rise at 0.075–0.1% (*p* < 0.05). However, correlation analysis revealed a significant negative association between the firmness and overrun after adding T40 and T60, yielding correlation coefficients of −0.905 and −0.926, respectively (*p* < 0.05). This indicated that the decrease in firmness was closely related to a higher overrun. The increased porosity and reduced solids in the foam weakened the structural support, which decreased the hardness. However, research suggested that lower firmness may result in a lack of creaminess during tasting, which in turn may lead to lower consumer preference [40,41]. This may be because whipped cream with higher firmness imparts a pleasant mouthfeel to the product, whereas lower firmness may result in a foamy texture. The results also showed that the effect of polysorbates on firmness was related to the chain length and saturation degree of the fatty acids. The saturated polysorbates (T20, T40, and T60) reduced firmness, while the unsaturated polysorbates (T80) increased the hardness at high concentrations. This may be attributed to the fact that the unsaturated fatty acid chains of T80 contribute to the induction of interfacial crystallization, which further promotes partial coalescence and strengthens the supporting function of the network skeleton [39].

#### 3.4.5. Microstructure at the End of Stirring

**Figure 13:**
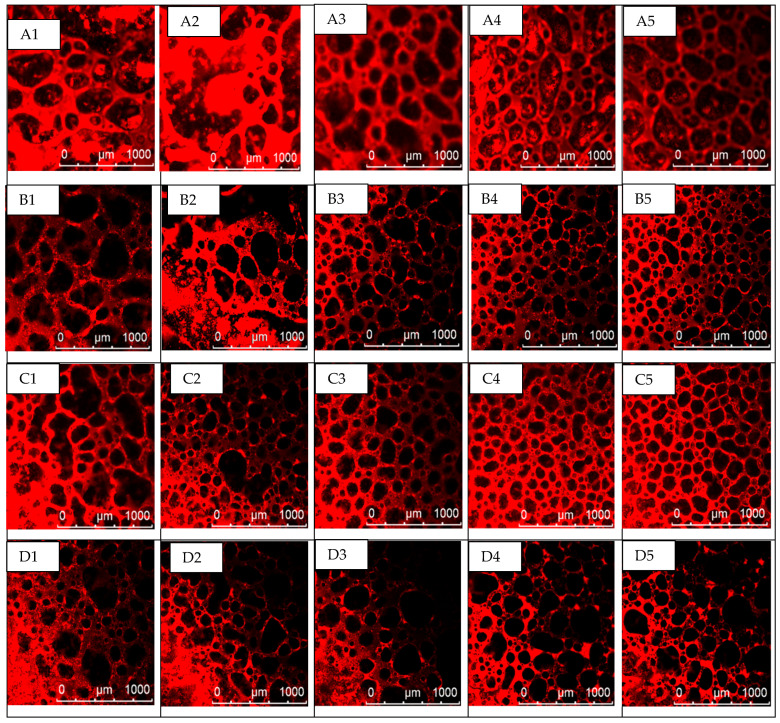
The fat network structure of the RDC emulsions after whipping with different polysorbate types and concentrations. (**A**–**D**) The laser confocal images of the whipped cream endpoints after adding T20, T40, T60, and T80, respectively; 1–5 represent added concentrations of 0%, 0.025%, 0.05%, 0.075%, and 0.1%, respectively. The red signals in the images denote fat, while the black circular areas signify air bubbles.

**Figure 14:**
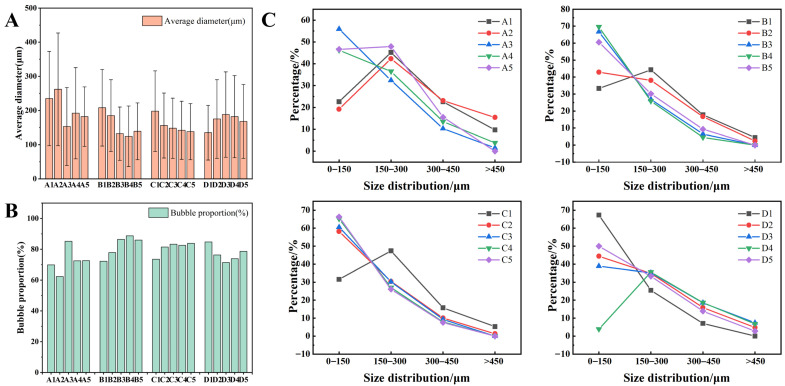
Average diameter (**A**), bubble percentage (**B**), and size distribution (**C**) based on the microstructure of the samples. The sample numbers correspond to those in Figure 12.

The textural properties of the foam system after aeration are closely related to its internal structure. A dense, uniform foam structure helps to improve structural stability. Figure 13 shows the microstructures of the different samples. Significant differences were evident between the effects of the four polysorbates on the microstructure. Higher T60 and T40 concentrations substantially reduced the bubble size, with a more uniform bubble distribution and an overall increase in bubble count. Analysis of the average bubble diameter (Figure 14A) and bubble proportion (Figure 14B) indicated that the average bubble diameter of samples T40 and T60 declined as the concentration increased. The average bubble diameter of group T60 (C1–C5) decreased from the initial 198 μm to 138 μm at a high concentration, while that of group T40 (B1–B5) declined from 208 μm to 124.5 μm. Furthermore, the proportion of small bubbles (0–150 μm) in both sample groups increased with the concentration, reaching 70% at a high concentration, which significantly exceeded the 30% in the low-concentration group. This explains the significant increase in the aeration expansion rate of the emulsion. The heterogeneity in bubble size and spatial distribution is directly governed by how the interfacial properties of fat globules modulate the partial coalescence rate. Moderate interfacial elasticity (exhibited by T40 and T60) allows uniform attachment of fat globules to air bubble surfaces during the whipping process, which facilitates the formation of a continuous and robust fat film, consequently reducing bubble size and enhancing their spatial distribution uniformity. Conversely, excessively low interfacial strength (exhibited by T20) induces rapid coalescence of fat globules into large fat agglomerates, preventing their uniform coverage on air bubble surfaces. However, no significant changes were evident in the bubble size and distribution of T20 and T80 after increasing the added concentrations. As shown in Figure 14, the average bubble diameters of T20 and T80 remained relatively stable at varying concentrations. As the concentration increased, the bubble diameters of the T20 group remained consistent in a range of 182–262 μm, while the T80 group showed a slight increase. The proportion of small bubbles was less than 60%, indicating no significant concentration-dependent changes, consistent with the observed microstructures of both samples. Furthermore, at different concentrations, the bubble proportion in the T20 sample was lower than in other samples, which corresponded to its lower overrun. Even at the same concentration, the polysorbates exhibited differing characteristics. Notably, the foam bridges in the T20 emulsion system were wider than those of T40, T60, and T80, which enhanced the cohesion and structural integrity of the foam [10]. This accounts for the lower serum loss observed in the T20 sample.

### 3.5. Discussion

The results of this study highlight the influence of polysorbate emulsifiers on the composition and rheological properties of the fat globule interface, as well as their impact on the stability and aeration capacity of cream mixtures. At the microscopic level, the regulatory effect of polysorbates on aerated emulsion systems is a dynamic process involving diffusion, competitive adsorption, and complex interactions with milk proteins. During homogenization, these small molecules rapidly adsorbed onto the oil–water interface, significantly reducing the initial interfacial tension. Rapid adsorption during the diffusion phase played a decisive role in the particle size during emulsion formation, significantly reducing the globule size and ultimately improving emulsion stability. However, during adsorption, competition between the polysorbates and interfacial proteins substantially decreased the interfacial protein concentration, which was consistent with the interfacial effects of other emulsifiers [42]. The weakening of covalent bonds, hydrophobic interactions, and disulfide bonds between the proteins significantly decreased the elastic modulus of the mixed interfacial layer from 46.2 mN/m to less than 20 mN/m, possibly further increasing fat globule sensitivity to shear behavior [23]. Therefore, polysorbates significantly reduced the whipping time since the high-fat globule sensitivity to shear behavior facilitated their rapid aggregation, resulting in the formation of a foam structure supported by a fat globule network. With the further decrease in interfacial strength (<10 mN/m), no further significant shortening of whipping time was observed, which indicates that an elastic modulus of 20 mN/m is an appropriate interfacial strength threshold. Notably, despite the considerably shorter whipping time, the overrun of the sample increased substantially. This may be because the presence of unadsorbed polysorbates in the liquid phase increases the foaming properties of the emulsion. The increase in the overrun at higher polysorbates concentrations validates this hypothesis.

From a quantitative perspective, various polysorbates exhibit noticeable differences in their ability to balance stability and aeration. While all the polysorbates significantly increased the shear sensitivity of the fat globules, the relatively high interfacial tension and protein concentrations in T40 and T60 possibly contributed to the static stability of the fat globules, consequently aiding emulsion stability. However, under strong mechanical action, they can still rapidly undergo partial aggregation to form a supporting framework. Furthermore, the lower interfacial adsorption capacity resulted in more unadsorbed polysorbates in the liquid phase, potentially enhancing bubble stability and significantly increasing overrun. Contrarily, the lower interfacial tension and protein concentrations in T20 and T80 resulted in the oversensitivity of the fat globules, raising the risk of aggregation even in static environments and increasing emulsion instability. Moreover, high T80 concentration (0.75–0.10%) increased firmness while improving overrun, possibly due to more thorough partial fat-globule aggregation.

## 4. Conclusions

This study systematically compared the effects of four polysorbates (T20, T40, T60, and T80) with different structures on the interfacial properties, microstructure, and macroscopic quality of recombined whipped cream in a concentration range of 0.025% to 0.1%. The results showed that the polysorbates significantly reduced interfacial tension through competitive adsorption with milk proteins at the oil–water interface, which reduced the particle size and improved emulsion stability. The influence of polysorbates is closely related to their molecular structure. An increase in the fatty acid carbon chain length (T20, T40, T60) decreased the substitution ability of the interfacial proteins, while increasing both the interfacial protein concentration and the fat globule size. The introduction of unsaturated double bonds (T60, T80) facilitated competitive polysorbate adsorption, which reduced the particle size. The reduction in interfacial proteins significantly decreased the mechanical strength of the interfacial layer, increasing the shear sensitivity of the fat globules and significantly reducing the whipping time. With increasing polysorbate dosage, despite the continuous decrease in interfacial protein content, no further reduction in whipping time was observed, demonstrating that the attenuation of interfacial strength imposes a limit on the shortening of whipping time. Furthermore, the marked increase in overrun reduces the structural density and supporting strength of the foam network, consequently resulting in a significant rise in serum loss. Meanwhile, proteins desorbed from the interface may adsorb at the gas–liquid interface and contribute to bubble stabilization, thus a linear correlation exists between serum loss and interfacial protein concentration. Future research should continue to investigate the synergistic effect of thickeners and polysorbate to improve product stability while ensuring aeration performance.

## Data Availability

The original contributions presented in this study are included in the article and Supplementary Materials; further inquiries can be directed to the corresponding author.

## Supplementary Materials

The following supporting information can be downloaded at: https://www.mdpi.com/article/10.3390/foods15111878/s1, Figure S1: The effect of the polysorbate type and concentration on the crystallization peak temperature of the RDC emulsions. (A) T20. (B) T40. (C) T60. (D) T80.

## Abbreviations

The following abbreviations are used in this manuscript:

RDC	Recombined whipped cream
Tween20	T20
Tween40	T40
Tween60	T60
Tween80	T80
