# Supplementary material for: Effects of Different Polysorbates (Tween) on Recombined Whipped Cream: Interfacial Properties, Stability, and Aeration Properties
Source: Foods. 2026 May 26;15(11):1878. doi: 10.3390/foods15111878 (PMC13257020; doi:10.3390/foods15111878)
Supplement: Supplementary file 1 [file foods-15-01878-s001.zip › foods-4297168-supplementary.pdf]

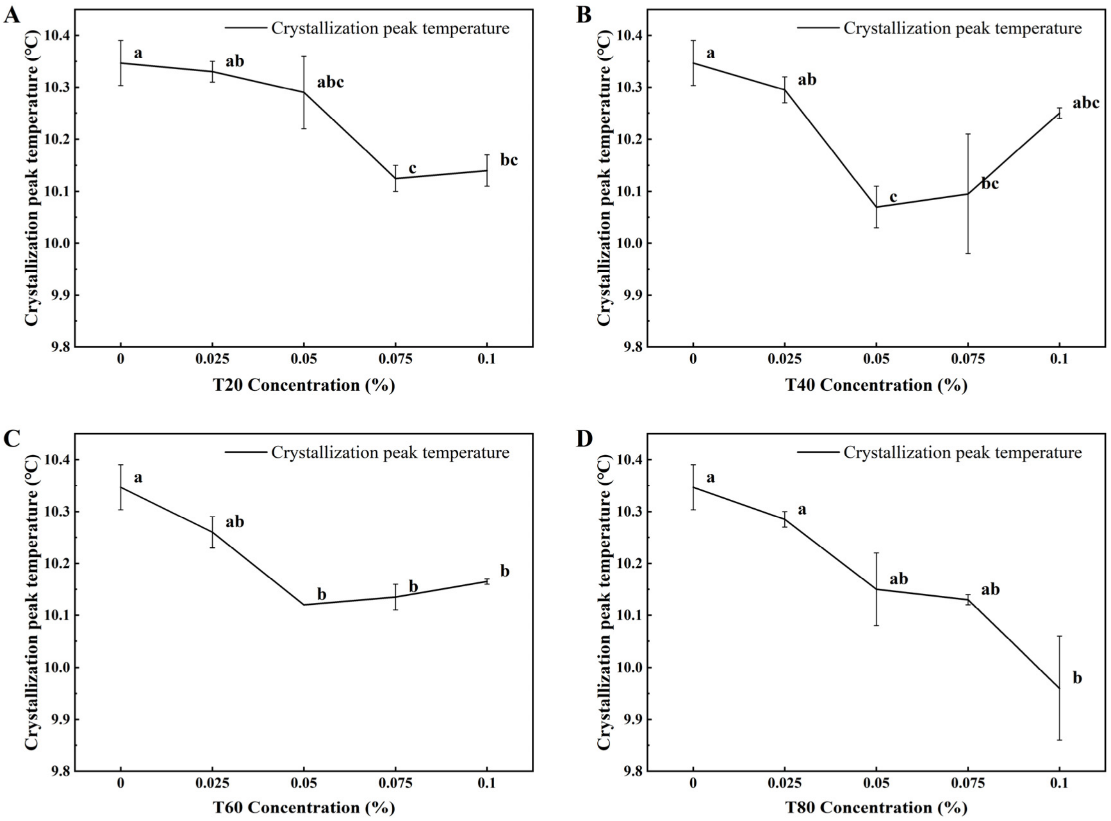

Figure S1. The effect of the polysorbate type and concentration on the crystallization peak temperature of the RDC emulsions. (A) T20. (B) T40. (C) T60. (D) T80
